# The transmittable through stinging microbiota differs between honeybees and wasps: a potentially greater microbial risk of the wasp sting for humans

**DOI:** 10.1007/s10123-023-00332-6

**Published:** 2023-02-08

**Authors:** Ioanna Gkitsaki, Alexandros Papachristoforou, Sofia Michailidou, Nikolaos Karamvalis, Ioannis Iliadis, Dimitra Graikini, Christina Sakarikou, Evangelos Tsoukis, Anagnostis Argyriou, Efstathios Giaouris

**Affiliations:** 1 Laboratory of Food Microbiology and Hygiene, Department of Food Science and Nutrition, School of the Environment, University of the Aegean, 81400 Myrina, Lemnos Greece; 2 Department of Food Science and Technology, School of Agriculture, Aristotle University of Thessaloniki, 57001, Thermi, Thessaloniki Greece; 3 Institute of Applied Biosciences, Centre for Research and Technology Hellas (CERTH), 57001, Thermi, Thessaloniki Greece; 4 Present address: Department of Animal Production and Sciences of the Food, Faculty of Veterinary Medicine, Zaragoza University, 50013 Zaragoza, Spain; 5 Microbiology Department of Lemnos Diagnostic Laboratory Medical S.A., 81400 Myrina, Lemnos Greece; 6 Laboratory of Molecular Genetics and Functional Genomics, Department of Food Science and Nutrition, School of the Environment, University of the Aegean, 81400 Myrina, Lemnos Greece

**Keywords:** Hymenoptera stinging, Honeybees, Wasps, Microbiota, Bacterial pathogens, Infection

## Abstract

The present research investigated whether accidental contact through stinging with honeybees, wasps, and hornets could represent a microbial hazard for humans. It has been previously suggested that such contact may transmit pathogens causing infections that could even be fatal for some susceptible individuals. Stinging simulation experiments were performed in the lab with live insects collected from the environment in Lemnos Island (north-eastern Greece), while different selective agar media targeting some clinically important bacteria (i.e., *Staphylococcus aureus*, *Streptococcus pyogenes*, *Enterococcus faecalis/faecium*, and *Pseudomonas aeruginosa*) were used as substrates for microbial recovery and identification. Results revealed none of the target pathogenic bacterial species in the honeybee samples, with bacilli, staphylococci, and micrococci dominating their surveyed microbiota. However, most of the suspect colonies isolated from wasps and hornets belonged to important hygienic indicators (i.e., enterococci, *Proteus mirabilis*, and coliforms), implying possible contact of these insects with fecal origin materials. To sum up, the microbiota that may be transmitted to humans through stinging appears to differ between honeybees and wasps/hornets, while the isolation from the latter samples of some other important opportunistic pathogens, such as *Enterobacter* spp. and *Klebsiella* spp., also known for multidrug resistance, could be an additional reason of concern.

## Introduction

Hymenoptera is a large order of insects, including members that are capable of stinging, such as bees, wasps, hornets, yellow jackets, and ants (Branstetter et al. 2018). Among them, bees are the dominant pollinators of angiosperms all over the world with more than 20,000 species described to date. These vary broadly in traits, such as nesting habitat, diet, and social behavior (Hedtke et al. 2013). More specifically, honeybees are social insects belonging to the *Apis* genus and live in well-organized communities. Besides their key beneficial role as pollinators in agriculture, they also provide several valuable products to humans, such as honey, beeswax, bee pollen, bee bread, royal jelly, and propolis (Martinello and Mutinelli 2021). These products are known for their plethora of functional (bioactive) properties and have been used by humans, mainly for nutrition and/or medicinal purposes, since ancient times (Cornara et al. 2017; Kurek-Górecka et al. 2020).

Although honeybees are beneficial to humans, they do still pose some danger due to their painful and venomous stings. Such exposure of humans to bee stinging dates back over 7000 years, when humans started to provide bee populations with artificial hives to harvest their honey and wax, or for pollination purposes (Pucca et al. 2019). However, most honeybees are not hostile toward humans or other animals and attack only when they are frightened and to defend their hives against intruders. *Apis mellifera* is the species that is mostly responsible for human envenoming in Europe (Pucca et al. 2019). Honeybees have barbed stingers that together with the venom sac are pulled out of the bee’s abdomen and remain in the victim’s skin following stinging; the insect dies shortly afterwards (Fitzgerald and Flood 2006). In contrast, wasps and hornets, both members of the Vespidae family, do not have barbed stingers and can sting multiple times without dying. They tend to be more aggressive than bees, stinging even without feeling threatened (Zirngibl and Burrows 2012).

Fortunately, most Hymenopteran stings are self-limiting (temporary) events, provoking only mild symptoms (such as erythema, edema, and pain at the sting site) which resolve without treatment in a few hours. However, massive envenomation may cause death depending on the victim’s age, body weight, number of stings, health, and immune status of the victim (Fitzgerald and Flood 2006). It has been roughly calculated that the European honeybee injects 147 μg of venom per sting, whereas most wasps inject about 17 μg of venom per sting, while the median lethal dose (LD_50_) of bee venom varies between 2.8 and 3.5 mg per kilogram of human body weight (Fitzgerald and Flood 2006). Therefore, a non-allergic person weighing 60–70 kg presents a 50% chance of death upon being stung by 1000–1500 bees, although deaths caused by many fewer stings have also been recorded (Pucca et al. 2019). Nevertheless, most deaths related to Hymenopteran stings are the result of immediate hypersensitivity reactions causing anaphylaxis. Such life-threatening anaphylactic reactions typically occur within 10 min of the sting and are not dose dependent or related to the number of stings (Adams et al. 2022).

Although bee venom is usually associated with pain resulting from the local inflammation in humans when stung by bees, during recent decades, venom has been applied for medicinal purposes because of its known antimicrobial and other therapeutical properties (Carpena et al. 2020; El-Seedi et al. 2020). Thus, bee venom through sting acupuncture is extensively used in apitherapy (Zhang et al. 2018). This alternate therapy relies on the usage of honeybee products, most importantly bee venom, for the treatment of several human diseases including neurodegenerative ones (such as Parkinson’s and Alzheimer’s), as well as some types of cancer (Oršolić 2012; Wehbe et al. 2019). For this reason, the venom is introduced into the human body by manual injection or directly via bee stings. However, with the latter, it is speculated that any bacteria or other microorganism found on either insects’ body or sting could be inoculated under the human epidermis during stinging. This may explain some reports of both local and disseminated bacterial infections after accidental bee stings (Anderson et al. 1995; Klug et al. 1982; Richardson and Schmitz 1997; Shahar and Frand 1979). Insect bites have also been reported to result in myocutaneous mycoses (Kontoyiannis et al. 2019). Viral infections following bee stings have also been reported (Can et al. 2021). Although all these infections are rarely fatal, there are also occasional reports of alarming deadly bacterial infections of susceptible human individuals following unintended honeybee stinging (Liang et al. 2021; Truskinovsky et al. 2001).

We therefore aimed to investigate whether accidental contact through stinging with honeybees could indeed represent a microbial hazard for humans. Wasps and hornets were also included in the experiments for comparative purposes. The latter are predaceous carnivores that live on other insects and sweet substances, such as sap and nectar, in contrast to honeybees that are herbivorous and live on nectar and pollen (Fitzgerald and Flood 2006). More specifically, our main goal was to detect whether some clinically important bacteria for human (i.e., *Staphylococcus aureus*, *Streptococcus pyogenes*, *Enterococcus faecalis/faecium*, and *Pseudomonas aeruginosa*) could be transmitted during the stinging procedure by these three important Hymenoptera insects (i.e., honeybees, wasps, and hornets). A series of four complementary stinging simulation experiments was performed.

## Materials and methods

### Collection of insects

A total of 288 honeybees (*Apis mellifera*), 252 wasps (*Vespula germanica*), and 36 hornets (*Vespa orientalis*) were collected from different environments, i.e., countryside, agricultural lands, and towns, in Lemnos Island, north-eastern Greece, during the period of April-May 2019. Special care was taken to increase the variety of the sampling sites (*n* = 9), by visiting various regions throughout the island, including both urban and rural areas. In addition, an equal number of insects of both categories (i.e., honeybees, vespids) were collected from each sampling site to minimize any unwanted bias that could arise due to the potential inhomogeneous environmental microbiota of each ecological niche. The numbers of each insect species inevitably differed due to the higher population density of honeybees and wasps compared to hornets at the sampling period (end of spring). The insects (*n* = 576) were caught and anesthetized by inhalation of carbon dioxide and directly transported to the Laboratory of Food Microbiology and Hygiene (LFMH; Department of Food Science and Nutrition, Lemnos), where they were individually placed in sterile Eppendorf tubes and temporarily stored at 4 °C for the stinging simulation experiments later the same day (see next section).

### Stinging simulation experiments

**Fig. 1:**
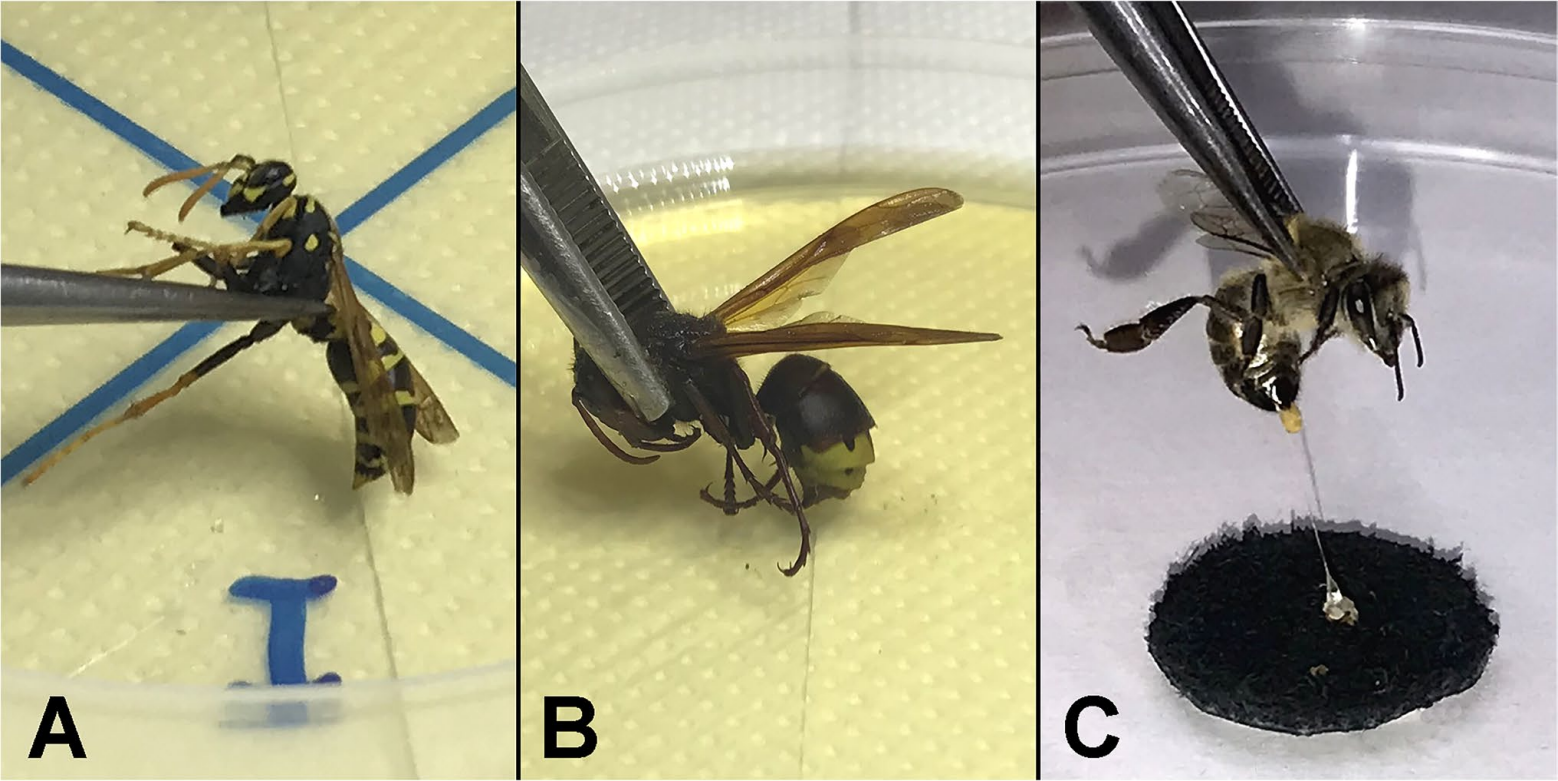
Stinging simulation by wasps *V. germanica* (**A**), hornets *V. orientalis* (**B**), and honeybees *A. mellifera* (**C**)

For each insect, experiments were conducted in four different but complementary ways, trying to thoroughly simulate the stinging procedure: (i) live insects were forced to sting directly (Fig. 1A and B) each nutrient substrate (x4); (ii) live insects were forced to sting sterilized leather as a human skin simulation (Fig. 1C), and immediately afterward, their sting apparatus was transferred to each nutrient substrate (x4); (iii) the third pair of insects’ legs (back legs), which are always in contact with insects’ victims during stinging incidents, were aseptically removed and were attached to each nutrient substrate (x4); and (iv) the whole anesthetized insect was inserted in a tube with saline solution (1 mL for honeybees and wasps, 5 mL for hornets; quarter-strength Ringer’s solution; Lab M, Heywood, Lancashire, UK), vortexed for 1 min at medium intensity (using a mini vortex mixer, VXMNAL; OHAUS Europe GmbH, Nänikon, Switzerland), and then an aliquot (100 μL) of the resulting suspension was transferred to each nutrient substrate and spread (x5; including the four selective agar media plus a general purpose one; see next section). It should be noted that the second approach (concerning leather stinging) was followed only for the honeybees since the sting apparatus of wasps and hornets is not removed from their body after stinging. For each replicate of each stinging simulation experiment (x4), a different insect was always used. This is because a given honeybee cannot sting twice, while in addition, a second successive stinging by a wasp or hornet would hinder our purpose which was mostly to check potential bacterial transfer from an “unused” sting. Αll experimental procedures were applied under aseptic conditions, using sterile forceps, and working near the flame of a Bunsen burner.

### Nutrient substrates used to isolate the target bacteria

Following each stinging simulation experiment (x4), four different types of selective agar media were used as the nutrient substrates to isolate the target bacteria, depending on the species. Thus, staphylococci were isolated on Baird Parker (BP) agar supplemented with Egg Yolk Tellurite (EYT), streptococci on blood agar supplemented with streptococcus selective supplement (COBA), enterococci on Kanamycin Aesculin Azide (KAA) agar, and pseudomonads on *Pseudomonas* Cephalothin, Fucidin, and Cetrimide (CFC) agar. BP, KAA, and CFC agars, together with their supplements, were all purchased from Lab M, while ready prepared streptococcal selective agar (COBA) plates (90 mm) were used that were provided by Oxoid (Thermo Fisher Scientific Inc, Microbiology, Perth, UK). Following inoculation of the plates, all the media were incubated aerobically at 37 °C for 24 h inside an incubator (Velp Scientifica™ FOC 215L; Fisher Scientific, Leicestershire, UK), except BP agar for which incubation was extended to 48 h (to obtain sufficiently grown colonies). Tryptone Soya Agar (TSA; Oxoid) was also surface inoculated as a general-purpose medium (i.e., in addition to the four selective agar media) in those stinging stimulation experiments where the whole anesthetized insects were individually subjected to vortexing (type iv; see “Stinging simulation experiments”). This was incubated at 30 °C for 72 h.

### Recovery of microbial isolates and identification of bacterial species

Following incubation of the plates, some representative colonies were isolated from all those showing visible growth. To achieve maximum possible diversity, for plates harboring up to three colonies, all colonies were recovered, whereas for plates with more colonies, a selection was made about which to isolate based on morphology (i.e., shape, size, color, and texture). More specifically, from all the different types of stinging simulation experiments, a total of 77 colonies were recovered, of which 30 were isolated from the honeybee experiments and the other 47 from the vespids. All isolates were sub-cultured through streaking on TSA to confirm their purity, and a freshly prepared colony of each one was also subjected to Gram staining. Only those isolates that were shown to be bacteria following microscopic observation (*n* = 64) were subjected to catalase reaction and then proceeded to identification, using one of the following five approaches: (i) PCR amplification of *16S rRNA* genes and sequencing using primer pairs either 63F/1542R or 27F/1518R and conditions as described by Galkiewicz and Kellogg (2008) and Giovannoni (1991), respectively (this was applied for 37 isolates from wasps and 5 isolates from hornets); (ii) PCR amplification of gyrase B (*gyrB*) gene and sequencing using primer pair UP-1/UP-2r and conditions as described by Yamamoto and Harayama (1995) (this was applied for 7 bacilli isolates from honeybees); (iii) PCR amplification of *rpoB* gene and sequencing using primer pair RpoB-F/RpoB-R and conditions as described by Hoffmann and Roggenkamp (2003) (this was applied for 5 *Enterobacter* spp. isolates from wasps); (iv) API® biochemical tests (using API^®^-Staph strips for 7 staphylococci/micrococci isolates and API^®^-20E strips for 1 *Enterobacter* sp. isolate, all from honeybees); and (v) matrix-assisted laser desorption-ionization time-of-flight mass spectrometry (MALDI-TOF MS) analysis as described by Jang and Kim (2018) (this was applied approach for 2 *Paenibacillus* spp. isolates from honeybees). These five different approaches were followed in our efforts to identify the species of each bacterial isolate since this was not possible for all the isolates through the *16S rRNA* gene sequencing.

All PCRs were done using the Kapa Taq PCR Kit (KK1016, 500 U; Kapa Biosystems, Wilmington, MA, USA) on a FastGene^®^ 96-well Ultracycler (FG-TC01 Gradient version; NIPPON Genetics EUROPE GmbH, Düren, Germany). All primer sequences that were here used are provided in Table 1. Before Sanger sequencing at the Institute of Applied Biosciences (CERTH, Thessaloniki, Greece), amplicons were purified using the NucleoSpin^®^ Gel and PCR Clean-up Kit (MACHEREY-NAGEL GmbH & Co. KG, Düren, Germany). API^®^ biochemical tests were done following the instructions of the manufacturer (bioMérieux SA, Marcy-l’Étoile, France), while MALDI-TOF MS analysis was executed on an external collaborating facility (Bioiatriki Healthcare Group, Athens, Greece). All identified isolates were long-term stored in the collection of LFMH at − 80 °C in cryovials containing Brain Heart Infusion (BHI) broth (Lab M), also supplied with 15% (v/v) glycerol.

**Table 1:** Primer sequences used in this study, together with their lengths (nt), GC contents (%), and references

Name	Sequence (5′-->3′)	Length (nt)	GC (%)	Reference
63F	CAG GCC TAA CAC ATG CAA GTC	21	52.4	Galkiewicz and Kellogg (2008)
1542R	AAG GAG GTG ATC CAG CCG CA	20	60.0	
27F	AGA GTT TGA TCM TGG CTC AG	20	47.5	Giovannoni (1991)
1518R	AAG GAG GTG ATC CAN CCR CA	20	55.0	
UP-1	GAA GTC ATC ATG ACC GTT CTG CAY GCN GGN GGN AAR TTY GA	41	51.2	Yamamoto and Harayama (1995)
UP-2r	AGC AGG GTA CGG ATG TGC GAG CCR TCN ACR TCN GCR TCN GTC AT	44	59.1	
RpoB-F	AAC CAG TTC CGC GTT GGC CTG G	22	63.6	Hoffmann and Roggenkamp (2003)
RpoB-R	CCT GAA CAA CAC GCT CGG A	19	57.9	

## Results and discussion

It is generally acknowledged that the microbial ecology of the environment of an insect, together with its nutritional and social habits, are parameters that shape its microbiome, both externally and internally. That microbiome influences in turn the overall insect health and productivity (including nutrient acquisition, metabolism, growth and development, and immune performance), as well as its potential to contaminate other hosts with the microorganisms it carries (Khan et al. 2020; Kwong et al. 2017). In this research, we focused on that part of the culturable microbiota of honeybees and vespids (including both wasps and hornets) that could be transmitted during the stinging procedure. This was done to investigate whether that could harbor pathogenic bacteria for human and more specifically whether some clinically important bacterial species (i.e., *S. aureus*, *S. pyogenes*, *E. faecalis/faecium*, and *P. aeruginosa*) could be transmitted during stinging. The choice of the four target bacterial species was based on their known involvement in potentially serious human infections. Thus, both *S. aureus* and *S. pyogenes* are part of the human skin microbiota and are also found in the upper respiratory tract (Egert and Simmering 2016; Byrd et al. 2018). While *S. aureus* is a frequent member of skin microbiota, with 20–30% of humans being long-term carriers, *S. pyogenes* is an infrequent pathogenic species being still carried by the 1–5% of the healthy individuals. Both species can cause important human infections, ranging from mild superficial skin infections to life-threatening systemic diseases (Cheung et al. 2021; Walker et al. 2014). *Enterococcus faecalis* and *E. faecium* are both commensal bacteria inhabiting the gastrointestinal (GI) tract of healthy humans and other mammals but can also cause life-threatening infections in immunocompromised individuals (García-Solache and Rice 2019). *P. aeruginosa* is also an important opportunistic pathogen recognized for its widespread occurrence, multidrug resistance against many antibiotics, and its frequent involvement in many hospital-acquired infections (Horcajada et al. 2019).

**Fig. 2:**
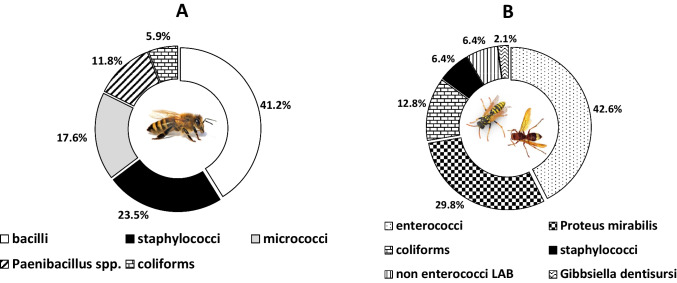
Ratios of bacterial isolates recovered from honeybees (**A**) and wasps and hornets (**B**)

To stimulate the stinging procedure, four complementary approaches were followed. Thus, the surfaces of both sting apparatus and back legs, which are always in contact with insects’ victims during stinging incidents, as well as the whole insect, were surveyed for their potential carriage of the target bacterial species through a culture-based procedure using mainly selective agar media. A total of 77 microbial isolates were successfully recovered, of which 13 were identified by microscopic observation as yeasts. It is worth noting that all these yeast isolates were recovered from the honeybee experiments. Νo yeast isolate was recovered from the wasp and hornet experiments, although these sugar-demanding eukaryotic microorganisms may also colonize these insects (Jimenez et al. 2017; Madden et al. 2018). Thus, all 47 microbial isolates recovered from the vespids were bacteria (5 were isolated from hornets, 42 from wasps), together with the 17 bacterial isolates recovered from honeybees. The identification results of these 64 bacterial isolates are presented in Table 2, while their presence ratios for each insect type (honeybees/vespids) are recorded separately in Fig. 2. Interestingly, for honeybees, none of the identified bacterial species belonged to any of the target clinically important bacteria. Honeybees were found to harbor mainly bacilli, staphylococci, and micrococci on their surveyed microbiota, with these three microbial groups consisting of the 82.4% of bacteria isolated from these insects (*n* = 14/17). On the other hand, enterococci (of which 9 *E. faecalis* isolates), *P. mirabilis*, and coliforms constituted the 85.1% of bacteria isolated from wasps and hornets (*n* = 40/47), suggesting the contact of these insects with fecal origin materials.

**Table 2:** Bacterial isolates recovered from honeybees (*A. mellifera*), wasps (*V. germanica*), and hornets (*V. orientalis*)

s/n	Isolate code	Bacterial genus/species	Gram staining	Catalase reaction	Insect species (host)	Stinging simulation experiment^a^	Nutrient substrate^b^	Identif. method^c^	NCBI accession number^d^
1	3	*Bacillus cereus* group	+	+	*A. mellifera*	iv	TSA	ii	OP009593
2	4	*Bacillus cereus* group	+	+	*A. mellifera*	iv	TSA	ii	OP009594
3	5	*Bacillus cereus* group	+	+	*A. mellifera*	iv	TSA	ii	OP009595
4	6	*Paenibacillus urinalis*	−	+	*A. mellifera*	iv	TSA	v	-
5	7	*Paenibacillus pabuli*	−	+	*A. mellifera*	iv	TSA	v	-
6	8Α	*Staphylococcus capitis*	+	+	*A. mellifera*	iv	TSA	iv	-
7	8Β	*Bacillus cereus* group	+	+	*A. mellifera*	iv	TSA	ii	OP009596
8	9	*Bacillus cereus* group	+	+	*A. mellifera*	iv	TSA	ii	OP009597
9	10	*Bacillus cereus* group	+	+	*A. mellifera*	iv	TSA	ii	OP009598
10	14	*Enterobacter cloacae*	−	+	*A. mellifera*	ii	CFC	iv	-
11	15	*Micrococcus luteus*	+	+	*A. mellifera*	ii	COBA	iv	-
12	18	*Staphylococcus cohnii*	+	+	*A. mellifera*	i	BP	iv	-
13	22	*Staphylococcus warneri*	+	+	*A. mellifera*	iii	BP	iv	-
14	27	*Micrococcus luteus*	+	+	*A. mellifera*	iii	COBA	iv	-
15	28	*Bacillus cereus* group	+	+	*A. mellifera*	iii	COBA	ii	OP009599
16	31	*Staphylococcus warneri*	+	+	*A. mellifera*	i	ΒP	iv	-
17	32	*Micrococcus luteus*	+	+	*A. mellifera*	i	COBA	iv	-
18	33	*Enterococcus sp.*	+	−	*V. germanica*	iii	TSA	i	ON994583
19	34	*Enterococcus mundtii*	+	−	*V. germanica*	iii	TSA	i	ON994584
20	35	*Enterococcus sp.*	+	−	*V. germanica*	iii	TSA	i	ON994585
21	36	*Enterococcus sp.*	+	−	*V. germanica*	iii	TSA	i	ON994586
22	37	*Enterococcus sp.*	+	−	*V. germanica*	iii	TSA	i	ON994587
23	38	*Staphylococcus epidermidis*	+	+	*V. germanica*	iv	ΒP	i	ON994588
24	39	*Enterococcus sp.*	+	−	*V. germanica*	iii	KAA	i	ON994589
25	40	*Enterococcus sp.*	+	−	*V. germanica*	iii	KAA	i	ON994590
26	41	*Enterococcus mundtii*	+	−	*V. germanica*	iii	COBA	i	ON994591
27	42	*Enterococcus mundtii*	+	−	*V. germanica*	iii	COBA	i	ON994592
28	44	*Proteus mirabilis*	−	+	*V. orientalis*	iv	TSA	i	ON994593
29	46	*Proteus mirabilis*	−	+	*V. orientalis*	iv	TSA	i	ON994594
30	47	*Gibbsiella dentisursi*	−	+	*V. orientalis*	iv	TSA	i	ON994595
31	48	*Enterococcus faecalis*	+	−	*V. orientalis*	iv	KAA	i	ON994596
32	49	*Staphylococcus sp.*	+	+	*V. orientalis*	i	KAA	i	ON994597
33	50	*Staphylococcus sp.*	+	+	*V. germanica*	iv	TSA	i	ON994598
34	53	*Enterobacter cancerogenus*	−	+	*V. germanica*	iv	TSA	iii	OP009588
35	54	*Enterococcus faecalis*	+	−	*V. germanica*	iv	TSA	i	ON994599
36	55	*Enterobacter cancerogenus*	−	+	*V. germanica*	iv	CFC	iii	OP009589
37	56	*Enterobacter cancerogenus*	−	+	*V. germanica*	iv	CFC	iii	OP009590
38	57	*Enterobacter cancerogenus*	−	+	*V. germanica*	iv	CFC	iii	OP009591
39	58	*Enterobacter sp.*	−	+	*V. germanica*	i	CFC	iii	OP009592
40	59Α	*Enterococcus faecalis*	+	−	*V. germanica*	i	KAA	i	ON994600
41	59Β	*Klebsiella sp.*	−	+	*V. germanica*	i	KAA	i	ON994601
42	60	*Proteus mirabilis*	−	+	*V. germanica*	iii	CFC	i	ON994602
43	61	*Proteus mirabilis*	−	+	*V. germanica*	iii	CFC	i	ON994603
44	62	*Proteus mirabilis*	−	+	*V. germanica*	iii	CFC	i	ON994604
45	63	*Proteus mirabilis*	−	+	*V. germanica*	iii	CFC	i	ON994605
46	64	*Proteus mirabilis*	−	+	*V. germanica*	iii	CFC	i	ON994606
47	65	*Proteus mirabilis*	−	+	*V. germanica*	iii	CFC	i	ON994607
48	66	*Proteus mirabilis*	−	+	*V. germanica*	iii	CFC	i	ON994608
49	67	*Proteus mirabilis*	−	+	*V. germanica*	iii	CFC	i	ON994609
50	68	*Proteus mirabilis*	−	+	*V. germanica*	iii	COBA	i	ON994610
51	69	*Enterococcus gallinarum*^†^	−	+	*V. germanica*	iii	COBA	i	ON994611
52	70	*Lactococcus lactis*^*†*^	+	−	*V. germanica*	iii	COBA	i	ON994612
53	71	*Enterococcus faecalis*	+	−	*V. germanica*	iii	COBA	i	ON994613
54	72	*Proteus mirabilis*	−	+	*V. germanica*	iii	COBA	i	ON994614
55	73	*Enterococcus gallinarum*^*†*^	+	−	*V. germanica*	iii	COBA	i	ON994615
56	74	*Lactococcus lactis*	+	−	*V. germanica*	iii	COBA	i	ON994616
57	75	*Enterococcus faecalis*	+	−	*V. germanica*	iii	COBA	i	ON994617
58	76	*Enterococcus faecalis*	+	−	*V. germanica*	iii	KAA	i	ON994618
59	77	*Enterococcus faecalis*	+	−	*V. germanica*	iii	KAA	i	ON994619
60	78	*Lactiplantibacillus plantarum*	+	−	*V. germanica*	iii	KAA	i	ON994620
61	79	*Proteus mirabilis*	−	+	*V. germanica*	iii	KAA	i	ON994621
62	81	*Enterococcus faecalis*	+	−	*V. germanica*	iii	KAA	i	ON994622
63	82	*Proteus mirabilis*	−	+	*V. germanica*	iii	KAA	i	ON994623
64	83	*Enterococcus faecalis*	+	−	*V. germanica*	iii	KAA	i	ON994624

^a^See “Stinging simulation experiments”

^b^see “Nutrient substrates used to isolate the target bacteria”

^c^see “Recovery of microbial isolates and identification of bacterial species”

^d^accession numbers of nucleotide sequences at National Center for Biotechnology Information (NCBI) databases (https://www.ncbi.nlm.nih.gov/)

^†^The primer pair 27F/1518R was used for the amplification and sequencing of the *16S rRNA* genes

Regarding honeybee’s microbiota, species belonging to the *Bacillus* genus are ubiquitous in nature and especially in soil and dust where they can differentiate themselves into oval endospores, being able to remain in this metabolically dormant state for years (Checinska et al. 2015). The species *Bacillus anthracis*, *B. cereus*, and *Bacillus thuringiensis* are phylogenetically closely related and together with some others that are less well studied (e.g., *Bacillus mycoides*, *Bacillus pseudomycoides*, *Bacillus cytotoxicus* etc.) belong to the so-called *B. cereus* group which contains species with pathogenic potential (Ehling-Schulz et al. 2019). Thus, *B. anthracis* is the etiologic agent of anthrax, a disease with a severity that varies depending on the host and route of infection. This can infect all mammals, some birds, and possibly even reptiles, but it primarily affects ruminants because of their frequent environmental exposure to that pathogen (Pilo and Frey 2018). Humans may be infected by ingesting spores found in foods (such as meat from infected animals), but today, most naturally acquired human cases of anthrax are cutaneous infections which are provoked following contact with infected animals or their products. *B. cereus* is commonly recognized as a foodborne pathogen, being able to produce toxins both upon its growth in foods and also inside the human gut, while strains of this species can also cause localized wound and eye infections, as well as systemic disease particularly in immunosuppressed individuals, intravenous drug users, and neonates, due to their ability to produce several tissue-destructive exoenzymes (Enosi Tuipulotu et al. 2021). *B. thuringiensis* strains are commonly known as entomopathogens, being able to produce potent species-specific insecticidal toxins, and for this reason have been commercialized for use as biopesticides in agriculture and for developing genetically modified cultivars (Palma et al. 2014). However, there are also reports documenting the ability of this microorganism to cause various infectious diseases in immunocompromised human individuals due to its ability to produce various virulence factors acting against mammalian cells (Celandroni et al. 2014). It should be noted that the differentiation of the species of the *B. cereus* group is really challenging (Ehling-Schulz et al. 2019), while in our case, this was not possible by either of the two approaches we followed (full sequencing of both the *16S rRNA* and *gyrB* genes).

The three *Staphylococcus* species recovered from honeybees (*S. capitis*, *S. cohnii*, and *S. warneri*) are all coagulase-negative (CoN) that are not normally dangerous to healthy people as skin commensals. However, all these have been implicated in infective endocarditis and bacteremia, both consisting of serious infections in immunocompromised individuals (Diaconu et al. 2019; Soldera et al. 2013; Thakker et al. 2021), while in neonates, they are included in the most common endemic nosocomial pathogens (Michels et al. 2021). This is in accordance with the general belief that CoN could be dangerous to sensitive population groups, such as the neonates, elderly, and immunocompromised, when these bacteria succeed to enter the body (Natsis and Cohen 2018). The same is also true for *Micrococcus luteus*, an obligate aerobic catalase-positive Gram-positive bacterium that can also cause infections in susceptible people (Martín Guerra et al. 2019; Seifert et al. 1995) and has been shown to survive in oligotrophic environments for extended periods of time (Greenblatt et al. 2004).

The two *Paenibacillus* isolates recovered from honeybees (i.e., *P. urinalis* and *P. pabuli*) belong to a genus with species that were formerly included in *Bacillus*. Like the latter, this contains spore-former species that are isolated from a variety of environments, most of them being found in soil and often associated with plant roots where they can promote plant growth via nitrogen fixation, phosphate utilization, iron acquisition, and phytohormone production (Grady et al. 2016). Some species (such as *Paenibacillus larvae*, *Paenibacillus apiarius*, and *Paenibacillus glabratella*) are pathogens to honeybees and other invertebrates; others are opportunistic pathogens for humans, rarely tending to cause infections to immunocompromised people, with *P. urinalis* being the first time isolated from a human urine sample in 2008 (Roux et al. 2008).

Bacteria of the *Enterobacter* genus belonging to the Enterobacteriaceae family are facultative anaerobic, Gram-negative, and widely distributed in nature and encountered in various environmental habitats. To date, that genus comprises more than twenty species that are found in soil, water, sewage, and plants (as either endophytic or phytopathogens for various plant species) and are also commensals of the animal and human gut (Davin-Regli et al. 2019). Alarmingly, in recent decades, *Enterobacter* spp. have emerged as important nosocomial pathogens mainly for immunocompromised patients hosted in intensive care units (ICUs). Indeed, these pathogens are included in the so-called ESKAPE group (*E. faecium*, *S. aureus*, *Klebsiella pneumoniae*, *Acinetobacter baumannii*, *P. aeruginosa*, and *Enterobacter* species), which comprises bacterial species that are leading etiological agents of resistant and life-threatening nosocomial infections (Chang et al. 2022). *E. cloacae* was the sole species isolated from the honeybees (one isolate), while five other *Enterobacter* isolates were recovered from wasps, of which the four belonged to *E. cancerogenus* species. Like other *Enterobacter*, *E. cloacae* is ubiquitous in terrestrial and aquatic environments and is also an important nosocomial (opportunistic) pathogen that can be responsible for bacteremia, endocarditis, septic arthritis, osteomyelitis, and several other infections. The skin and the GI tract are the sites through which this bacterium is most commonly acquired, while it is worth noting that *Enterobacter* spp. in general are responsible for approximately 5% of nosocomial bacteremia cases (Mezzatesta et al. 2012).

Twenty enterococci isolates were recovered from vespids comprising the 42.6% of bacteria isolated from these insects, of which nine were *E. faecalis*, three *E. mundtii*, and two *E. gallinarum*. It was not possible to identify the other six *Enterococcus* spp*.* isolates to species level (through the *16S rRNA* gene sequencing approach here followed). In general, enterococci are Gram-positive bacteria that can survive in conditions unfavorable to other bacteria (García-Solache and Rice 2019) and are frequently employed as hygienic indicators, with their presence in foods and water denoting fecal contamination (Boehm and Sassoubre 2014). These are commensals of the GI tract of many metazoans, from insects to humans. Although they normally do not cause disease once found in that habitat, they can become pathogenic when they infect sites outside the intestine (Yuen and Ausubel 2014). Recently, enterococci have gained attention as important nosocomial pathogens, with *E. faecalis* and *E. faecium* being the two species causing the majority of human enterococcal infections (Yuen and Ausubel 2014). Alarmingly, both species present intrinsic resistance to several common antibiotics (such as cephalosporins and aminoglycosides) but can also readily acquire further resistance to other antibiotics, due to plasticity in their genomes (García-Solache and Rice 2019). More specifically, *E. faecalis* is well adapted to persist in multiple niches within the mammalian host and can quickly adjust its metabolism to survive in new environments, something which, together with the arsenal of strategies it possesses to confront innate and adaptive immune mechanisms, enable it to cause infections in many sites within the host including the GI tract, urinary tract, wounded epithelium, heart, and blood (Kao and Kline 2019).

*Proteus mirabilis* was the most abundant species that was isolated from vespids, with a total of 14 isolates recovered from wasps and hornets (consisting of the 29.8% of the isolates). It is a Gram-negative rod-shaped proteolytic bacterium belonging to the Enterobacteriaceae family and in humans is an opportunistic pathogen well-known for its swarming motility and ability to produce urease, often causing catheter-associated urinary tract infections (UTIs) (Armbruster et al. 2018). *P. mirabilis* can be encountered in various environments, including soil, water, and sewage, but is generally a commensal of the GI tracts of humans and animals. However, although commensal, *P. mirabilis* is equipped with a shocking arsenal of virulence factors and is thus capable of causing a variety of human infections, with bacteremia and sepsis provoked by this bacterium presenting a high mortality rate. Alarmingly, the existence of multidrug-resistant isolates further complicates the therapeutic treatment options (Girlich et al. 2020). In general, *Proteus* spp. bacteria, like enterococci and coliforms, are often regarded as indicators of fecal pollution of the environments, with human and animal feces being probably their source in those environments (Drzewiecka 2016).

The third most isolated bacterial group from vespids was coliforms which are also considered strong indicators of fecal contamination (Mishra et al. 2018). Thus, five *Enterobacter* spp. isolates and one *Klebsiella* sp. isolate were recovered from wasps (12.8% of the isolates). Like most other coliforms, *Klebsiella* species belong to the Enterobacteriaceae family. They are found everywhere in nature and considered opportunistic pathogens able to cause a wide range of diseases, particularly among those with weakened immune systems, such as neonates, elderly, and immunocompromised individuals, including pneumonia, UTIs, bloodstream infections, and sepsis (Bengoechea and Sa Pessoa 2019). Alarmingly, the increasing isolation of multidrug-resistant strains, such as those belonging to the *K. pneumoniae* species, significantly hampers the therapeutic options for the treatment of these infections (Navon-Venezia et al. 2017).

Three staphylococci, of which one is *S. epidermidis* (also included in CoN species previously reported); three lactic acid bacteria (LAB), including two *Lactococcus lactis* and one *Lactiplantibacillus plantarum*; and *Gibbsiella dentisursi* were the remaining bacterial species isolated from wasps and hornets. In general, LAB comprises a group of generally recognized as safe (GRAS) bacteria that are found in decomposing plants, human GI, and vaginal microflora and used for centuries in food fermentations (Raman et al. 2022). A few members of this group are also included in the core gut microbiota of social bees (Kwong and Moran 2016). *G. dentisursi* is a largely unknown, rod-shaped, Gram-negative species of the Enterobacteriaceae family that was first isolated from the oral cavity of a bear in Japan (Saito et al. 2012).

## Conclusions

Although the target pathogenic bacterial species (i.e., *S. aureus*, *S. pyogenes*, *E. faecalis/faecium*, and *P. aeruginosa*) were not recovered from honeybees, the isolation from these insects of some important opportunistic pathogens (such as *E. cloacae* and CoN staphylococci), together with potentially pathogenic species of the *B. cereus* group, does not in theory exclude the possibility of a honeybee sting being fatal to someone with a weakened immune system. However, to the best of our knowledge, such opportunistic pathogens have never been described as etiological agents of infections caused following bee stinging. In addition, it would be probably rather impossible to not isolate at all opportunistic bacterial pathogens from an organism living free in the environment, such as the honeybee, also considering that most bacteria, if not all microorganisms, may cause infections under certain (opportunistic) conditions. On the other hand, most of the suspect colonies isolated from wasps and hornets belonged to important hygienic indicators (i.e., enterococci, *P. mirabilis*, and coliforms), suggesting that these insects were in contact with fecal origin materials. The simultaneous isolation from these insects of some other important opportunistic pathogenic bacterial species, such as *Enterobacter* spp. (10.6% of isolates) and *Klebsiella* spp., also known for multidrug resistance, might cause serious concern. Future studies could be conducted employing a larger sample of insects and preferably metagenomic approaches to try to fully unravel the total (i.e., not only the culturable) sting microbiota of the most important Hymenoptera species, such as the ones already performed on the gut microbiota of some of them.

## Data Availability

The data presented in this study are available on reasonable request from the corresponding author.
