# Correction to: The transmittable through stinging microbiota differs between honeybees and wasps: a potentially greater microbial risk of the wasp sting for humans

**DOI:** 10.1007/s10123-023-00342-4

**Published:** 2023-02-24

**Authors:** Ioanna Gkitsaki, Alexandros Papachristoforou, Sofia Michailidou, Nikolaos Karamvalis, Ioannis Iliadis, Dimitra Graikini, Christina Sakarikou, Evangelos Tsoukis, Anagnostis Argyriou, Efstathios Giaouris

**Affiliations:** 1 Laboratory of Food Microbiology and Hygiene, Department of Food Science and Nutrition, School of the Environment, University of the Aegean, 81400 Myrina, Lemnos Greece; 2 Department of Food Science and Technology, School of Agriculture, Aristotle University of Thessaloniki, 57001 Thermi, Thessaloniki Greece; 3 Institute of Applied Biosciences, Centre for Research and Technology Hellas (CERTH), 57001 Thermi, Thessaloniki Greece; 4 Present Address: Department of Animal Production and Sciences of the Food, Faculty of Veterinary Medicine, Zaragoza University, 50013 Zaragoza, Spain; 5 Microbiology Department of Lemnos Diagnostic Laboratory Medical S.A., 81400 Myrina, Lemnos Greece; 6 Laboratory of Molecular Genetics and Functional Genomics, Department of Food Science and Nutrition, School of the Environment, University of the Aegean, 81400 Myrina, Lemnos Greece

**Correction to: International Microbiology**

**https://doi.org/10.1007/s10123-023-00332-6**

The authors regret that an unnecessary sentence* was embedded at the beginning of the “Results and discussion” section.

* “We refer to 3 different parameters:

(i) microbial ecology of the environment.

(ii) nutritional habits.

(iii) social habits”

The original article has been corrected. The authors apologize for any inconvenience caused.

## Data Availability

Corresponding authors Alexandros Papachristoforou and Efstathios Giaouris are responsible for the inclusion of any availability statement.

